# Idiopathic intracranial hypertension presenting with isolated unilateral facial nerve palsy: a case report

**DOI:** 10.1186/s13256-019-2060-5

**Published:** 2019-04-19

**Authors:** Ahmad Samara, Dana Ghazaleh, Brent Berry, Malik Ghannam

**Affiliations:** 1 An-Najah National University, Nablus, Palestine; 2 University of Minnesota, Minneapolis, MN USA; 3 Neurology Department, University of Minnesota, Minneapolis, MN USA

**Keywords:** IIH, CN VII palsy, Rare association, Anatomical correlation

## Abstract

**Background:**

Idiopathic intracranial hypertension, also known as pseudotumor cerebri, is a disorder characterized by increased intracranial pressure of unclear pathogenesis in the absence of other structural and obstructive lesions that is predominantly, although not exclusively, seen in obese women of childbearing age. Patients with idiopathic intracranial hypertension commonly present with a headache, transient visual obscurations, and intracranial noises with some cranial nerves occasionally involved, most commonly CN VI. We report idiopathic intracranial hypertension presenting with isolated complete unilateral facial nerve palsy, as the sole cranial nerve involved, which is a presentation rarely reported in the literature.

**Case presentation:**

A 40-year-old Hispanic woman with a history of obesity and hypertension presented to our emergency department complaining of bifrontal headache for 3 days associated with nausea, vomiting, transient visual disturbances, and a picture of right-sided cranial nerve VII palsy. Her neurologic examination including other cranial nerves was otherwise normal, but a fundus examination revealed bilateral grade II papilledema. Imaging studies ruled out structural and obstructive lesions as possible causes of her symptoms and lumber puncture results were unremarkable except for an increased opening pressure. She was then started on prednisone and acetazolamide. Two days later, she reported a dramatic improvement in both headache and facial nerve palsy.

**Conclusions:**

Idiopathic intracranial hypertension should be suspected in obese young women presenting with headache and transient visual complaints and some cranial nerve abnormalities. Idiopathic intracranial hypertension is a diagnosis of exclusion and imaging studies should always be performed to rule out other structural and obstructive lesions. In this case report, we aimed to draw attention to the possibility of idiopathic intracranial hypertension presenting with unilateral cranial nerve VII palsy as the only cranial nerve involved, which needs a high index of suspicion by clinicians. The mechanisms of cranial nerve VII palsy in idiopathic intracranial hypertension are not well understood and prompt further investigation.

## Background

Idiopathic intracranial hypertension (IIH), also known as pseudotumor cerebri, is a disorder characterized by increased intracranial pressure (ICP) of unclear pathogenesis, which implies the absence of intracranial mass lesions or clear cerebrospinal fluid (CSF) outflow obstruction [1, 2]. IIH is predominantly seen in overweight and obese women of childbearing age and has also been associated with high-dose vitamin A derivatives, tetracycline, and estrogen-progestin oral contraceptives [3]. Patients affected by IIH commonly present with headache (92%), transient visual obscurations (72%), and intracranial noises (60%) [4].

IIH can also be associated with single or multiple cranial nerve (CN) palsies, with 39–59% of the patients having some sort of CNs deficit. The most common CN palsy is that of CN VI, documented in 12% of adults and 9–48% of children [5–7]. Less frequently, palsies of other CNs can be encountered, including CN III, IV, VII, IX, and XII [5]. In a limited number of cases, CN VII (facial nerve) palsy has been reported in association with IIH and other CN and/or CNs involvement [8] and, in even fewer cases, as isolated unilateral CN VII palsy.

In light of this, we present one of the rare cases of IIH presenting with isolated complete unilateral CN VII (facial nerve) palsy as the sole CN involved. This alerts clinicians to suspect IIH in high-risk patients who present with isolated unilateral CN VII palsy.

## Case presentation

A 40-year-old Hispanic woman, with a history of obesity, a body mass index (BMI) of 32, and hypertension, presented to our emergency department (ED) complaining of squeezing bifrontal headache for 3 days. Her headache started gradually, had a progressive course, and was associated with nausea, vomiting, dizziness, transient visual disturbances in her right eye, and a feeling of both her ears being clogged. A day prior to the presentation, she started to feel numbness and weakness of the right side of her face, along with an inability to close her right eye properly. She denied having diplopia, loss of vision, photophobia, tinnitus, or any feeling of weakness, numbness, or tingling in other locations of her body. She had no history of migraine headaches, tick bite, or any recent illness or fever. She was not taking oral contraceptive pills at the time.

On initial evaluation, she was hemodynamically stable and afebrile. On neurological examination, she was fully alert and oriented, and had fluent speech and intact comprehensive abilities. There were no signs of meningeal irritation. CN testing revealed: 3–4 mm pupils that were equal in size and reactive to light and accommodation; intact extraocular movements with no nystagmus, saccadic movement or skew; and full visual fields. No signs of abducens nerve palsy were present. However, there was facial asymmetry evident by right lower facial droop, weaker right eye closure, and limited ability to raise the right eyebrow. Facial sensation, on the other hand, was equal on both sides, with a strong jaw opening and a midline tongue of good power. In addition, shoulder shrug was symmetrical, and hearing was intact. A fundus examination revealed bilateral grade I–II papilledema. The rest of her neurological examination, including motor function, sensation, reflexes, coordination, and gait analysis, was within normal limits.

**Fig. 1:**
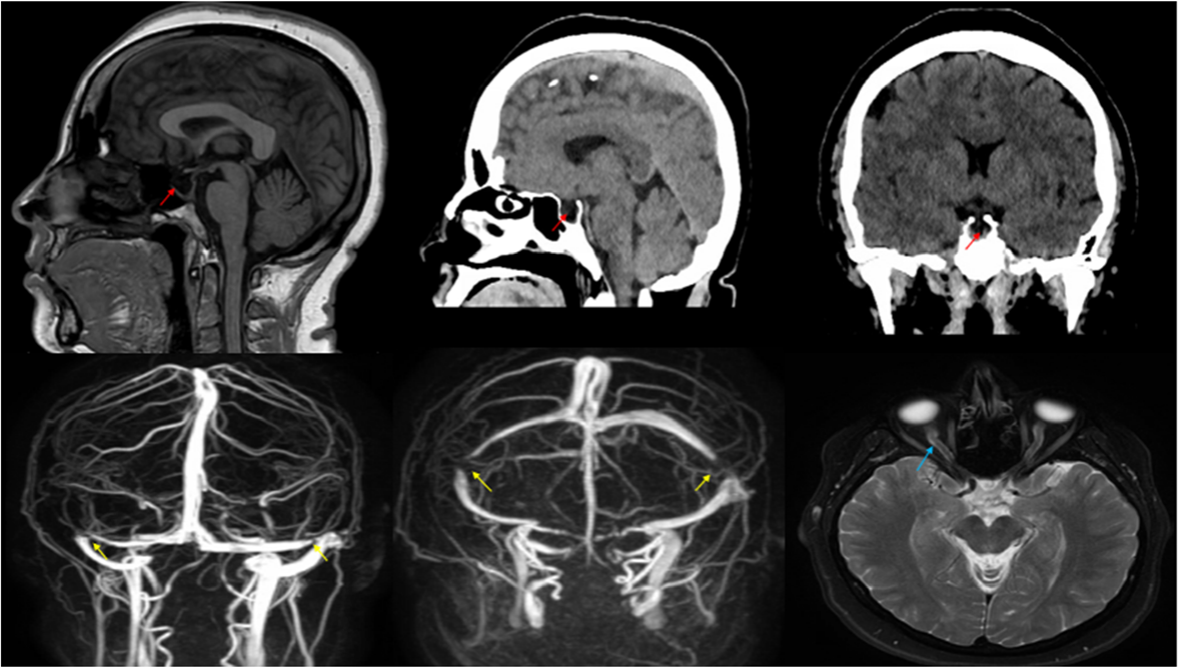
*Top*: A computed tomography scan of the head in the top middle picture showed pontomedullary hypodensity, and a partially empty sella turcica, noting the top first one on the left is brain MRI (red arrows). *Bottom*: Brain magnetic resonance imaging with magnetic resonance venography revealed a stenosis in the lateral aspect of the transverse sinus (*yellow arrows*), and perioptic nerve sheath distention which is consistent with papilledema (*blue arrow*)

She underwent a computed tomography (CT) scan of her head that showed some right-sided pontomedullary hypodensity. Brain magnetic resonance imaging (MRI) with magnetic resonance venography (MRV) revealed a stenosis in the lateral aspect of the transverse sinus, a partially empty sella turcica, and a picture of mild papilledema, findings consistent with ICP (Fig. 1). A lumbar puncture (LP) produced CSF with an opening pressure of 28 cm, which is above the limit of the reference interval. The cytological and chemical findings of the LP were otherwise within normal limits: white blood cells (WBCs) 2, lymphocytes 100%, protein 24, red blood cells (RBCs) 13, and glucose 58. She was initially treated with intravenously administered 25 mg diphenhydramine and 10 mg metoclopramide along with 500 ml intravenous normal saline 0.9% (IVF) and her headache subsequently subsided. She was also started on prednisone 60 mg daily for 5 days and 500 mg of acetazolamide twice daily. Two days later, she reported a dramatic improvement in both the headache and the facial nerve palsy. A week later, she attended our clinic for a right facial nerve examination, which was completely normal.

## Discussion

We present the case of an obese 40-year-old woman complaining of a bifrontal headache and features of unilateral complete facial nerve palsy. No signs of other CNs involvement were evident. A neurological examination was otherwise normal, but fundus examination was significant for bilateral minimal-to-low degree papilledema. Imaging studies including CT scan and MRI/MRV ruled out structural and obstructive lesions and showed only signs of ICP, leaving the diagnosis of IIH as the most probable cause of this patient’s symptoms. This diagnosis was further supported by the marked improvement upon receiving acetazolamide and prednisone therapy.

IIH predominantly affects obese women with an incidence of 11.9 per 100,000 per year in this group compared to its incidence in the general population at 1.56 per 100,000 per year [9]. IIH fits our patient diagnosis, however, the unusual unilateral facial nerve involvement is atypical and makes consideration of other brain lesions legitimate, namely brainstem stroke and other structural lesions. These disorders were eventually ruled out by the absence of supporting features on the imaging studies and the clinical presentation as well.

The pathophysiology of facial nerve palsy in IIH is not entirely understood and no satisfactory hypotheses seem to exist. Most studies have described it as a false localizing sign [10–12], possibly resulting from increased ICP. The extra pressure is believed to exert traction forces on the extra-axial facial nerve [6, 13, 14]. The theory is quite acceptable in cases of combined sixth and seventh nerves dysfunction, due to the well-known fact that the abducens nerve is the most susceptible of all CNs to high ICP explained by its long course. In contrast, the anatomy of the seventh nerve makes it less prone to such an effect. From the motor nucleus in the pons, facial nerve fibers wrap around the sixth nerve nucleus forming the facial colliculus and then run a relatively short course before reaching the petrous part of the temporal bone where it resides, somehow protected from pressure effects [11]. Given these facts, it is extremely unusual to have an isolated facial nerve palsy from high ICP, with the abducens nerve usually spared. The possibility of this association being merely coincidental in our patient should not be completely refuted. However, many factors would suggest otherwise, such as symptoms of facial nerve palsy occurred in the same time period as those of IIH [15].

One case made a relation between facial nerve palsy and IIH based on an enlargement of the fallopian canal of the facial nerve at the geniculate nucleus that acted as a predisposing factor for the clinical presentation [8]. In our patient, imaging studies showed no such anomaly. Another study explained the association by hypervitaminosis A as a common cause of both facial nerve palsy and IIH [16], which is not the case in our patient.

Many imaging findings have been traditionally described in IIH. These include perioptic nerve sheath distention, vertical buckling of optic nerve, globe flattening, optic nerve head protrusion, and an empty sella turcica. A retrospective study by Hingwala *et al.* described the frequencies of each of these signs in IIH, as shown in Table 1, and concluded that only optic nerve head protrusion and globe flattening can help in differentiating IIH from other secondary causes of increased ICP [17].

**Table 1:** Occurrence of imaging findings in idiopathic intracranial hypertension

Finding	%
Perioptic nerve sheath distention	95.2
Empty sella turcica	76.2
Optic nerve head protrusion	71.4
Globe flattening	71.4
Vertical buckling of optic nerve	61.9

(Adapted from Hingwala *et al*., [17])

Other conditions that were considered in the differential diagnosis of this case include neurosarcoidosis (NS) and Lyme disease affecting the nervous system. CN deficits are the most common manifestation of NS, and CN VII is the most commonly involved CN in NS. However, sarcoidosis affecting only the nervous system is extremely rare, with an annual incidence of less than 0.2 per 100,000. MRI (with and without intravenously administered gadolinium) is the imaging modality of choice in the evaluation of NS, with the most common finding of this condition being the involvement of the leptomeninges (including the arachnoid membrane and pia mater), which is seen as linear enhancement along the brain contours and extending into the cortical sulci [18]. Lyme disease can also affect both the central nervous system (CNS) and the peripheral nervous system (PNS) with CN VII being the most commonly involved CN in PNS. However, CNS Lyme disease is practically excluded in the presence of a completely normal CSF profile [19], and the fact that Lyme disease involving only the PNS cannot explain our patient’s CNS manifestations. The lack of a history of tick exposure or fever made Lyme disease even less likely in this patient. Having our patient fulfilling all criteria of IIH as described in its latest revision by Friedman *et al.* in 2013 [20], with the lack of enough diagnostic evidence to support the other two differential diagnoses, these two diagnoses were abandoned in favor of the more plausible diagnosis of IIH.

In addition, Bell’s palsy coinciding with or occurring on top of IIH was another possible but complicated explanation of this unusual presentation considering the relatively low incidence of each of these two conditions to occur separately, which makes the chance of them coinciding unlikely, especially knowing that CN VII palsy can occur in association with IIH, albeit rarely. Besides, Bell’s palsy cannot explain this patient’s headache or the increase in ICP on its own. Also, because rapid reversing of the CN palsy with lowering of the ICP is required to associate the palsy with IIH [7], the clinical course of our patient, by fulfilling this condition, supported the view that our patient’s CN VII palsy was caused by IIH. However, the currently recommended treatment of Bell’s palsy also consists of the use of prednisone preferably within 72 hours of the onset of symptoms [21].

The definite pathophysiologic mechanisms behind IIH are still uncovered, but many theories have been proposed which conventionally involved CSF production and absorption and cerebral venous pressure elevation. Radio-isotopic studies have suggested an increased arachnoid resistance to CSF efflux in IIH, especially in obese women [22], whereas three-dimensional contrast-enhanced MRI studies showed that most cases of IIH involved stenosis along the transverse–sigmoid sinus junction which may result from an intrinsic abnormality in the sinus wall (such as an arachnoid granulation, scar tissue, or septation) and appears as a focal region of stenosis, or from an extrinsic process − in this case, the compression caused by the elevated ICP – that gives a more tapered appearance [23].

Our patient was treated with acetazolamide and a short course of prednisone. Acetazolamide, a potent carbonic anhydrase inhibitor, works by decreasing the production of CSF and is widely accepted as the preferred medical therapy for IIH [1, 2]. Although the use of corticosteroids is considered controversial in IIH, their use in combination with acetazolamide can be beneficial in patients with concerns of visual loss, with the controversy being mainly about the long-term use of corticosteroids for fears of their serious side effects, such as weight gain and salt retention, which might aggravate the problem [4]. In cases in which the medical therapy fails, certain surgical procedures can be sought out in order to stop the progression of visual loss. These procedures include optic nerve sheath fenestration, stenting the transverse sinus, and placing a shunt for CSF diversion [2].

After our patient was started on medical therapy as mentioned above, she was scheduled to see the interventional neuroradiologist to be assessed for possible transverse sinus stenting in the future. Transverse sinus stenting reduces both cerebral venous pressure and ICP and therefore improves symptoms and signs in patients with IIH in selected situations [1]. A recent prospective trial by Dinkin and Patsalides [23] concluded that transverse sinus stenting may be safely considered an alternative therapy for patients with IIH requiring surgical management, but it is not yet evident that any surgical procedure for IIH is superior to another.

A very few cases of IIH with facial nerve palsy as the sole CN involved have been previously reported in the literature. Chutorian *et al.* [15] reported three such cases, in two girls and one boy, their ages ranged from 11 to 14 years, all three had unilateral CN VII palsy, and the duration of their symptoms ranged from 3 to 8 weeks. They were all treated with serial LPs. Another case of IIH associated with unilateral CN VII palsy was reported by Zachariah *et al.* [24] in a 29-year-old woman who was treated with prednisone and acetazolamide and had her symptoms for 4 weeks’ duration. Kiwak and Levine [25] reported a case of IIH associated with diplegic CN VII palsy as the only CN involved in a 28-year-old woman who was treated with lumboperitoneal shunt, whereas Selky *et al.* [14] reported a similar case in a 17-year-old girl for whom both medical and surgical therapy failed and she ended up with permanent symptoms. Capobianco *et al.* [10] reported two cases of IIH with unilateral CN VII palsy but one was also associated with CN VI palsy and required optic nerve sheath decompression. Cases of IIH associated with CN VII palsy along with other CNs involvement are relatively more common. Davie *et al.* [26] reported a case of 25-year-old woman with IIH and both CN VII and CN VI involvement who was treated with LP. Another similar case, but with additional CN III involvement was reported by Agarwal *et al*. [27] and was treated with LP, dexamethasone, and glycerol syrup. Another three cases of IIH with both CN VII and CN V involvement were reported by Bakshi *et al*. [28], Couch *et al*. [29], and Hagberg and Sillanpaa [30] and were treated with lumboperitoneal shunt, LP, and LP and dexamethasone, respectively. Snyder and Frenkel [31] reported a case of IIH with unilateral CN VII palsy and ophthalmoplegia in a 25-year-old woman who was treated with LP and dexamethasone and had the symptoms for 10 days. A case of IIH associated with unilateral CN VII palsy and enlargement of the fallopian canal on CT scan and MRI was reported by Brackmann and Doherty [8] in an 8-year-old boy who was treated with acetazolamide, prednisolone, and serial LPs. Another case of IIH with unilateral CN VI and contralateral CN VII palsy was reported by Soroken *et al.* [6] in a 13-year-old girl. She was treated with LP and acetazolamide and improved within 3 weeks of the treatment.

## Conclusions

IIH should be strongly suspected in obese young women presenting with headache and transient visual complaints. Some CN abnormalities can occasionally be present in patients with IIH. The diagnosis of IIH remains one of exclusion and imaging studies should always be performed to rule out other structural and obstructive lesions.

Here we aimed to draw attention to the possibility of IIH presenting with isolated unilateral CN VII palsy as the only CN involved, which has very rarely been described. We reviewed the available literature for such association to better understand the pathophysiology and the clinical significance of this injury. The association between isolated CN VII palsy and IHH needs high index of suspicion by clinicians and it is a diagnosis of exclusion. The mechanisms of CN VII palsy in IHH are not well understood and prompt further investigation.

## Abbreviations

BMI: Body mass index
CN: Cranial nerve
CNS: Central nervous system
CSF: Cerebrospinal fluid
CT: Computed tomography
ED: Emergency department
ICP: Increased intracranial pressure
IIH: Idiopathic intracranial hypertension
IVF: Intravenous normal saline 0.9%
LP: Lumbar puncture
MRI: Magnetic resonance imaging
MRV: Magnetic resonance venography
NS: Neurosarcoidosis
PNS: Peripheral nervous system
RBC: Red blood cell
WBC: White blood cell

## References

[1] Biousse V, Bruce BB, Newman NJ. Update on the pathophysiology and management of idiopathic intracranial hypertension. J Neurol Neurosurg Psychiatry. 2012;83(5):488–94. 10.1136/jnnp-2011-302029 PMC3544160 22423118

[2] Madriz Peralta G, Cestari DM. An update of idiopathic intracranial hypertension. Curr Opin Ophthalmol. 2018;29(6):495–502. 10.1097/ICU.0000000000000518 30169466

[3] Acheson JF. Idiopathic intracranial hypertension and visual function. Br Med Bull. 2006;79-80:233–44. 10.1093/bmb/ldl019 17242038

[4] Wakerley BR, Tan MH, Ting EY. Idiopathic intracranial hypertension. Cephalalgia. 2015;35(3):248–61. 10.1177/0333102414534329 24847166

[5] Wall M, George D. Idiopathic intracranial hypertension. A prospective study of 50 patients. Brain. 1991;114(Pt 1A):155–80. 1998880

[6] Soroken C, Lacroix L, Korff CM. Combined VIth and VIIth nerve palsy: Consider idiopathic intracranial hypertension! Eur J Paediatr Neurol. 2016;20(2):336–8. 10.1016/j.ejpn.2015.11.007 26653361

[7] Rangwala LM, Liu GT. Pediatric idiopathic intracranial hypertension. Surv Ophthalmol. 2007;52(6):597–617. 10.1016/j.survophthal.2007.08.018 18029269

[8] Brackmann DE, Doherty JK. Facial palsy and fallopian canal expansion associated with idiopathic intracranial hypertension. Otol Neurotol. 2007;28(5):715–8. 10.1097/01.mao.0000281801.51821.27 17667777

[9] Raoof N, *et al.* The incidence and prevalence of idiopathic intracranial hypertension in Sheffield, UK. Eur J Neurol. 2011;18(10):1266–8. 10.1111/j.1468-1331.2011.03372.x 21426442

[10] Capobianco DJ, Brazis PW, Cheshire WP. Idiopathic intracranial hypertension and seventh nerve palsy. Headache. 1997;37(5):286–8. 10.1046/j.1526-4610.1997.3705286.x 9195767

[11] Kearsey C, *et al.* Seventh nerve palsy as a false localizing sign in benign intracranial hypertension. J R Soc Med. 2010;103(10):412–4. 10.1258/jrsm.2010.100049 PMC2951170 20829324

[12] Larner A. False localising signs. J Neurol Neurosurg Psychiatry. 2003;74(4):415–8. 10.1136/jnnp.74.4.415 PMC1738389 12640051

[13] Tzoufi M, *et al.* Idiopathic intracranial hypertension and facial palsy: case report and review of the literature. J Child Neurol. 2010;25(12):1529–34. 10.1177/0883073810375849 20921567

[14] Selky AK, Dobyns WB, Yee RD. Idiopathic intracranial hypertension and facial diplegia. Neurology. 1994;44(2):357. 10.1212/wnl.44.2.357 8198627

[15] Chutorian AM, Gold AP, Braun CW. Benign intracranial hypertension and Bell’s palsy. N Engl J Med. 1977;296(21):1214–5. 10.1056/NEJM197705262962107 854059

[16] Obeid M, *et al.* Facial palsy and idiopathic intracranial hypertension in twins with cystic fibrosis and hypovitaminosis A. Pediatr Neurol. 2011;44(2):150–2. 10.1016/j.pediatrneurol.2010.10.002 21215918

[17] Hingwala DR, *et al.* Imaging signs in idiopathic intracranial hypertension: Are these signs seen in secondary intracranial hypertension too? Ann Indian Acad Neurol. 2013;16(2):229–33. 10.4103/0972-2327.112476 PMC3724081 23956571

[18] Terushkin V, *et al.* Neurosarcoidosis: presentations and management. Neurologist. 2010;16(1):2–15. 10.1097/NRL.0b013e3181c92a72 20065791

[19] Halperin JJ. Nervous system Lyme disease. Infect Dis Clin N Am. 2015;29(2):241–53. 10.1016/j.idc.2015.02.002 25999221

[20] Friedman DI, Liu GT, Digre KB. Revised diagnostic criteria for the pseudotumor cerebri syndrome in adults and children. Neurology. 2013;81(13):1159–65. 10.1212/WNL.0b013e3182a55f17 23966248

[21] Mooney T. Diagnosis and management of patients with Bell’s palsy. Nurs Stand. 2013;28(14):44–9. 10.7748/ns2013.12.28.14.44.e7979 24299386

[22] Orefice G, *et al.* Radioisotopic cisternography in benign intracranial hypertension of young obese women. A seven-case study and pathogenetic suggestions. Acta Neurol (Napoli). 1992;14(1):39–50. 1580203

[23] Dinkin MJ, Patsalides A. Venous Sinus Stenting in Idiopathic Intracranial Hypertension: Results of a Prospective Trial. J Neuroophthalmol. 2017;37(2):113–21. 10.1097/WNO.0000000000000426 27556959

[24] Zachariah SB, *et al.* Pseudotumour cerebri with focal neurological deficit. J Neurol Neurosurg Psychiatry. 1990;53(4):360–1. 10.1136/jnnp.53.4.360 PMC1014185 2341857

[25] Kiwak KJ, Levine SE. Benign intracranial hypertension and facial diplegia. Arch Neurol. 1984;41(7):787–8. 10.1001/archneur.1984.04050180113031 6743073

[26] Davie C, Kennedy P, Katifi HA. Seventh nerve palsy as a false localising sign. J Neurol Neurosurg Psychiatry. 1992;55(6):510–1. 10.1136/jnnp.55.6.510 PMC1014916 1619427

[27] Agarwal MP, Mansharamani GG, Dewan R. Cranial nerve palsies in benign intracranial hypertension. J Assoc Physicians India. 1989;37(8):533–4. 2621191

[28] Bakshi SK, *et al.* Facial nerve involvement in pseudotumor cerebri. J Postgrad Med. 1992;38(3):144–5. 1303420

[29] Couch R, Camfield PR, Tibbles JA. The changing picture of pseudotumor cerebri in children. Can J Neurol Sci. 1985;12(1):48–50. 10.1017/s0317167100046588 3978475

[30] Hagberg B, Sillanpaa M. Benign intracranial hypertension (pseudotumor cerebri). Review and report of 18 cases. Acta Paediatr Scand. 1970;59(3):328–39. 10.1111/j.1651-2227.1970.tb09012.x 5444713

[31] Snyder DA, Frenkel M. An unusual presentation of pseudotumor cerebri. Ann Ophthalmol. 1979;11(12):1823–7. 556136

